# Developing Value-Added Protein Ingredients from Wastes and Byproducts of Pulses: Challenges and Opportunities

**DOI:** 10.1021/acsomega.2c00414

**Published:** 2022-05-23

**Authors:** Asli Can Karaca, Michael T. Nickerson

**Affiliations:** † Department of Food Engineering, Istanbul Technical University, 34469 Istanbul, Turkey; ‡ Department of Food and Bioproduct Sciences, University of Saskatchewan, Saskatoon, Canada S7N 5A8

## Abstract

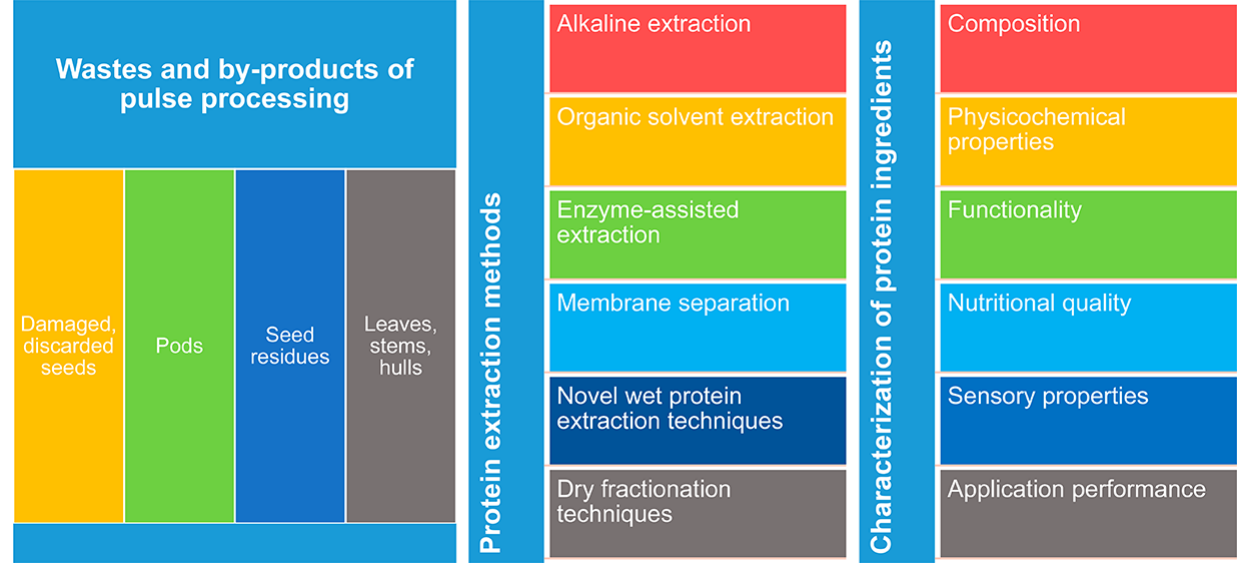

Wastes and byproducts of pulse processing carry a potential for utilization as raw materials for extraction of protein ingredients. This work is an overview of the extraction and fractionation techniques used for obtaining protein ingredients from wastes and byproducts of pulse processing, and it presents several characteristics of proteins extracted in terms of composition, nutritional properties, and functional properties. Several extraction methods have been applied to obtain protein ingredients from pulse processing wastes and byproducts. Each extraction technique is indicated to have significant effects on protein composition and functionality which could also affect the performance of proteins in different food applications. Versatile end product applications of protein ingredients obtained from pulse processing wastes and byproducts are yet to be discovered. Research is lacking on the limitations and improvement methods for using wastes and byproducts of pulses for protein extraction. This review provides insights into the possible applications of innovative extraction technologies for obtaining protein ingredients from wastes and byproducts of pulses. Further research has to focus on various modification techniques that can be applied to improve the functional, nutritional, and sensory properties of proteins extracted from pulse processing wastes and byproducts.

## 1 Introduction

Wastes and byproducts of pulse processing constitute a broad concept and include various different elements. Damaged pulse seeds discarded during harvesting and field processing are considered as residues or byproducts, whereas pods and other seed residues are separated during cleaning and splitting operations in industrial pulse processing. Moreover, leaves, stems, and empty pods are generated during canning, freezing, and/or drying processes applied to pulses.^1^ Beans, peas, and chickpeas constitute a significant portion of the pulses produced globally. Although there is limited information on the amount of wastes and byproducts of pulses generated, individual reports indicate that 5–25% of the legume crops initially harvested are separated as residue/waste.^1^ Moreover, the milling process applied to pulses is indicated to yield ∼25% byproducts including husk, powder, and broken, shriveled, and unprocessed seeds.^2^

Wastes and byproducts of pulse processing are considered as a sustainability problem in terms of environmental deterioration and are generally used as animal feed or substrate for biofuel production.^1^ However, they are rich in proteins and fibers which can be turned into value-added products. Many versatile ingredients including proteins, dietary fiber, starch, and phenolic compounds can be extracted from pulse wastes and byproducts. Extraction of proteins from plant-based food wastes and byproducts have been recently reviewed.^2−4^ Moreover, Tassoni et al.^1^ reviewed the extraction of various ingredients such as proteins, fibers, and other bioactive molecules from legume processing byproducts, residues, and wastes. The main goal of this mini-review is to cover the recent studies focusing on developing value-added protein ingredients from wastes of pulses. Special emphasis is given to the challenges associated with utilization of proteins extracted from pulse wastes and byproducts and some possible strategies for overcoming these challenges.

## 2 Extraction and Characterization of Protein Ingredients from Wastes and Byproducts of Pulses

### 2.1 Extraction and Fractionation Techniques

Wastes and byproducts of canning, freezing, and drying processes applied to pulses include a mixture of leaves, stems, empty pods, hulls, and discarded, dark, or spotted seeds.^1^ Valorization of these wastes and byproducts can be achieved through extraction of proteins by several wet and dry fractionation methods. Wet fractionation methods include alkaline extraction followed by isoelectric precipitation, organic solvent extraction, enzyme-assisted extraction, and membrane separation.^1^ Alkaline extraction followed by isoelectric precipitation is among the most commonly used techniques for extracting proteins from pulses. The process usually starts with solubilizing proteins at alkaline pH (8.0–11.0) far from their isoelectric point and is followed by precipitation of the solubilized proteins at the pH close to the isoelectric point (pH 4.5–4.6). Enzymes can be applied in protein extraction for the purpose of disruption of the cell wall integrity and for improving the protein extraction yield. However, enzyme-assisted extraction processes are indicated to have high operational costs and energy consumption. Membrane-based separation methods can be used as an alternative to the commonly applied isoelectric precipitation method since these processes can be operated under milder conditions and show a relatively higher yield of protein recovery. Furthermore, novel wet protein extraction techniques such as subcritical water extraction, reverse micelle extraction, and aqueous two-phase system extraction are attracting attention recently since those techniques are more cost-effective and environmentally friendly compared to traditional protein extraction methods.^2^ Dry fractionation methods including sieving and/or air classification techniques for separation of protein-rich and carbohydrate-rich fractions are applied to pulses on a commercial scale. A novel dry fractionation process based on electrostatic separation was also utilized for fractionation of pulse flours.^2^ Application of dry fractionation methods to protein extraction from wastes and byproducts of pulses is still limited.^1^ Any technique used for protein extraction from pulses can also be applied to wastes and byproducts of pulse processing; however, some modifications may be required to improve yield, protein quality, and functionality. The choice of the most appropriate extraction technique depends on the type and composition of the matrix, the scale of processing, and several other parameters related to the protein extracted such as the desired protein content, quality, and functionality, and the end product application intended. Findings of some of the recent studies focusing on extracting protein ingredients from wastes and byproducts of pulses are summarized in Table 1.

**Table 1:** Extraction of Protein Ingredients from Wastes and Byproducts of Pulse Processing

waste/byproduct utilized	fractionation method	outcome	ref
disease-damaged beans (*Phaseolus vulgaris* L.)	alkaline extraction followed by isoelectric precipitation	ACE-I-inhibitory activities of the peptides obtained from damaged beans were reported to be similar to those obtained from control.	(5)
chickpea and pea processing feedstocks	direct aqueous extraction and enzyme-assisted extraction	Direct aqueous extraction was reported to preserve protein integrity, whereas enzyme-assisted extraction resulted in relatively higher protein digestibility, determined by hydrolysis degree before and after digestion.	(6)
byproducts of milling of black gram (*Vigna mungo* L.)	dry fractionation	The milled fractions were indicated to be rich in proteins (12–42%), showed good antioxidant activity, and were suggested for nutraceutical applications.	(23)
byproducts of milling of moth bean (*Vigna aconitifolia* L.)	dry fractionation	The protein-rich fraction was indicated to be a good source of protein and minerals. Water and oil absorption capacities and foaming and emulsifying properties were found to be suitable for food applications.	(7)
pea (*Pisum sativum* L.) and broad bean (*Vicia faba* L.) pods	removal of pods via shelling	Pea and broad bean pods were indicated to contain considerable amounts of protein (11–14%), high amounts of dietary fiber (40–59%), and minerals.	(24)

### 2.2 Composition, Physicochemical Properties, and Functional Properties of Proteins Extracted

Composition and physicochemical properties are listed among the most important intrinsic factors affecting protein functionality. Physicochemical properties include surface hydrophobicity, net surface charge, molecular elasticity, interfacial properties, and viscosity in solution. Amino acid and polypeptide compositions and structural, physicochemical, and functional properties of pulse proteins are extensively studied. However, there is limited research on characterization of proteins extracted from wastes and byproducts of pulses. Hernandez-Alvarez et al.^5^ extracted proteins from anthracnose disease-damaged beans (*Phaseolus vulgaris* L.). The amino acid compositions and electrophoretic profiles of proteins extracted from disease-damaged beans were compared with those of control beans. It was reported that anthracnose disease resulted in minor changes in the amino acid profiles of bean proteins. Arg, Val, Thr, Ile, and Cys contents of proteins extracted from disease-damaged beans were found to be higher than those of control beans, whereas Asp + Asn, Tyr, Pro, and Ser contents were reported to be lower. Electrophoretic profiles of proteins extracted from disease-damaged and control beans were observed to be similar, where the molecular weights ranged from 15 to 200 kDa and the major band corresponded to phaseolin.^5^

In a recent study, Prandi et al.^6^ applied direct aqueous extraction and enzyme-assisted extraction techniques for protein extraction from chickpea and pea processing byproducts. Effects of the extraction method on the electrophoretic profile, degree of hydrolysis, free amino acid contents, and nutritional properties of proteins were investigated. Direct aqueous extraction using a neutral phosphate buffer (pH 7.2) and a 1:2 solid to liquid ratio was reported to be the most suitable technique for preserving protein integrity with a 1–5% degree of hydrolysis and a free amino acid content of <1%. The protein extraction method was found to affect the amino acid compositions of the proteins extracted from both chickpea and pea processing byproducts which was explained by extraction of different protein patterns with different methods. In addition to the amino acid composition, digestibility is another important factor affecting protein quality and bioavailability. Digestibility of proteins extracted with the enzyme-assisted method using specific proteases was found to be significantly higher than that of proteins extracted with the direct aqueous method according to a simulated gastrointestinal model.^6^

In another recent study, Kamani et al.^7^ investigated the composition and functional properties of the byproducts of milling of moth beans (*Vigna aconitifolia* L.). The amino acid profile of the protein-rich milled fraction (∼25% protein) was observed to be similar to that of moth bean flour. The protein-rich fraction was reported to show suitable functionality in terms of water and oil absorption capacities, foaming properties, and emulsifying properties. On the other hand, solubility of the protein-rich fraction (∼18%) was found to be lower compared to that of whole flour (∼25%).^7^

### 2.3 Promising End Product Applications

Protein ingredients extracted from wastes and byproducts of pulse processing find various applications in food and feed.^1^ Among food applications, snacks and bakery products are among the most commonly investigated end product applications for testing the performance of protein-rich ingredients obtained from byproducts of pulses.^8,9^

In addition to the wastes and byproducts generated during pulse processing, disease-damaged and frost-affected pulses were also investigated as potential sources of functional components and ingredients for novel food products. Hernandez-Alvarez et al.^5^ evaluated the potential of anthracnose disease-damaged beans (*P. vulgaris* L.) as a source of angiotensin-converting enzyme inhibitory (ACE-I) peptides. Anthracnose is a fungal disease which reduces yield and commercial value by damaging the seeds, resulting in significant economic losses. The authors extracted proteins from beans damaged by anthracnose disease with alkaline extraction followed by an isoelectric precipitation method, and regular beans were used as the control. Protein concentrates obtained were subjected to enzymatic hydrolysis with Alcalase 2.4 L and tested for the degree of hydrolysis and ACE-I activity for determining the potential antihypertensive effect of the hydrolysates. Although the nutritional profile of disease-damaged beans was found to be different from that of regular beans, no significant differences were observed in amino acid composition. Furthermore, the hydrolysis kinetics, electrophoretic profile, and ACE-I inhibitory activity of the peptides obtained from damaged beans were reported to be similar to those obtained from control beans. The authors suggested that disease-damaged beans could potentially be used as a raw material for the extraction of bioactive peptides.

In another recent study, Portman et al.^10^ investigated the nutritional profile of premium quality (grade1) and downgraded frost-affected lentils (*Lens culinaris* M.) to be used as ingredients in extruded foods. The reasoning behind the use of frost-damaged lentils was that the visual appearance of seeds would not be an essential quality parameter if the seeds were going to be used for protein extraction or production of novel food ingredients. The authors prepared composite wheat–lentil flour mixes using varying ratios of grade1 and frost-affected lentil flours (0–100%) and used them in a high-temperature–high-pressure extrusion process. Changes in the nutritional composition and phenolic profile of the samples from the extrusion process were determined. No significant differences were observed in the protein or carbohydrate contents of grade1 and frost-affected lentils. On the other hand, the extrusion process was found to result in a significant decrease in total protein content and maltose and glucose concentrations. Inclusion of lentil flour in the mix was reported to result in a significant increase in phenolic acids; however, the extrusion process was observed to reduce the concentration of phenolic acids. Overall, the nutritional compositions and functionalities of grade1 and frost-affected lentils were found to be similar. The authors suggested that frost-affected lentils could be utilized as a source of novel food ingredients and help reduce food waste and costs.

### 2.4 Aquafaba

The functional properties of aquafaba, the wastewater from the canning or cooking process of chickpeas, have gained considerable interest lately.^11^ Foaming, emulsifying, gelation, and thickening properties of aquafaba are utilized for developing many novel vegan foods.^12^ Current research on aquafaba is focusing on the optimization of the seed to water ratio and processing parameters for improved yield and functionality. The effects of several parameters including the chickpea cultivar, soaking temperature and duration, cooking temperature, pressure, duration, and pH on the yield, functional properties, and content of antinutritive compounds of aquafaba were investigated recently.^11,13−16^ Apart from chickpeas, other pulses including peas^17^ and lima beans^18^ were also evaluated for aquafaba production and applications. Standardization of processing methods and minimizing water and energy usage still remain as challenges in aquafaba production.^12^

## 3 Limitations and Improvement Methods for Using Wastes and Byproducts of Pulses for Protein Extraction

### 3.1 Increasing Protein Extraction Yield

Lower protein extraction yields observed in extraction processes from pulse waste and byproducts compared to whole pulse seeds may hinder the scaling up of the extraction processes and commercialization of the value-added ingredients obtained. Various strategies including utilization of novel extraction technologies and application of enzymes are used for improving extraction yields and functionalities of proteins obtained from pulse wastes and byproducts.^2^ Some of the novel techniques applied for improved protein extraction yield include ultrasound treatment, microwave-assisted extraction, and application of a pulsed electric field. Although these techniques have not yet found wide application in the extraction of proteins from pulse waste and byproducts, promising results were obtained for various other plant-based byproducts including rice bran, soy byproducts, and fruit seeds.^3^ Another strategy for increasing protein extraction efficiency is the application of enzymes such as cellulase and pectinase which release proteins attached to the polysaccharides in plant cells and increase protein extraction efficiency.^19^ Enzyme-assisted protein extraction processes have been widely applied to soybean residues; however, applications to pulse waste and byproducts are still scarce.^1^

### 3.2 Improving Protein Functionality

Due to their relatively poorer functionality compared to animal-based proteins and the presence of antinutrients and undesired flavors, utilization of plant-based proteins in food formulations can be challenging.^20^ Application of various modification techniques based on chemical, physical, and enzymatic processes is used as an effective strategy for improving protein functionality. Some examples from recent studies focusing on the modification of structural and physicochemical characteristics and improvement of functional properties of pulse proteins are indicated in Table 2. Any technique that has been found to be effective for the improvement of functional properties of proteins obtained from pulses can be applied to proteins extracted from wastes and byproducts of pulse processing as well.

**Table 2:** Some Examples from Recent Studies Focusing on Improvement of Pulse Protein Functionality

pulse protein	process applied	effects observed	ref
pea	hydrolysis with 11 proteolytic enzymes	changes within the molecular weight distribution depending on the degree of hydrolysis; improved solubility and foaming and emulsifying properties	(25)
pea	freeze–thaw cycling	improved water and oil absorption capacities and foaming and emulsifying properties	(26)
chickpea	hydrolysis with three proteases	changes in amino acid profile; decreased trypsin and chymotrypsin inhibitory activities; changes in protein quality depending on the degree of hydrolysis	(27)
fava bean	ultrasound-assisted alkaline treatment	dissociation of larger aggregates, increased surface hydrophobicity, decreased free sulfhydryl groups, improved solubility and emulsifying and foaming properties	(28)
mung bean	microwave-assisted phosphorylation	introduction of phosphate groups to the structure, alterations in secondary structure, increased electronegativity and solubility; improved water-holding and oil-binding capacities and foaming and emulsifying properties	(29)

Similar to pulse proteins, improvement of the functional properties of aquafaba is a current research topic as well. Using a response surface design, Lafarga et al.^14^ optimized the pH and boiling conditions for improved foaming and emulsifying properties of aquafaba. It was reported that lowering the pH of aquafaba from 6.5 to 3.5 and decreasing the chickpea to cooking water ratio from 1:5 to 1:1.5 resulted in improved foaming and emulsion capacity and stability. Color and sensory attributes of meringues and mayonnaises prepared using the aquafaba produced under the optimized conditions were reported to be comparable with those of the products prepared with egg proteins. Alsalman et al.^21^ also applied response surface methodology for the optimization of aquafaba functionality. The authors used an I-optimal mixture design to investigate the effects of chickpea to cooking water ratio and cooking time on yield, protein content, functional properties, and contents of antinutritional factors. Both the chickpea to cooking water ratio and the cooking time were found to be effective for aquafaba functionality. The optimum conditions for improved emulsifying and foaming properties, water and oil holding capacities, and minimized tannin and phytate contents were determined to be 1.5:3.5 chickpea to cooking water ratio and 60 min of cooking time. In another recent study, Meurer et al.^22^ applied ultrasound treatment for the improvement of the foaming and emulsifying properties of aquafaba. The authors reported that protein solubility and density were not affected by the ultrasound treatment. On the other hand, the foaming and emulsifying properties of aquafaba were improved with ultrasound treatment using higher power intensity.

## 4 Conclusion and Future Outlook

Utilization of wastes and byproducts of the pulse processing industry as raw materials for the extraction of value-added protein-rich food ingredients is gaining importance recently from a sustainability perspective. Novel extraction technologies have been found effective in improving protein extraction yield and functionality. However, there are still challenges that need to be addressed such as improvement of the nutritional and sensory qualities of proteins extracted from wastes and byproducts of pulses. Further research has to focus on effects of innovative extraction technologies on nutritional properties and minimization or masking of off-flavors. Modification methods applied to pulse proteins for improvement of functionality can also be applied to proteins extracted from pulse waste and byproducts. More studies are needed in the future in order to elucidate the potential of value-added protein ingredients for utilization in innovative functional products for the growing global market.
